# Knowledge, attitudes, and practice toward postoperative cognitive dysfunction among anesthesiologists in China: a cross-sectional study

**DOI:** 10.1186/s12909-024-05358-6

**Published:** 2024-04-01

**Authors:** Li Hu, Shuai Kang, Qiaoyi Peng, Erdan An, Jian Lu, Hao Yang, Hongmei Zhou, Bin Zhang

**Affiliations:** 1 Department of Anesthesiology, The Second Affiliated Hospital of Jiaxing University, Jiaxing, China; 2 Zhejiang Chinese Medical University, Hangzhou, China; 3 Department of Anesthesiology, Shanghai Pudong New Area People’s Hospital, Shanghai, China

**Keywords:** Knowledge, attitudes, and practice, Postoperative cognitive dysfunction, Anesthesiologists, China, Cross-sectional study

## Abstract

**Background:**

To investigate the knowledge, attitudes, and practice (KAP) toward postoperative cognitive dysfunction (POCD) among anesthesiologists in China.

**Methods:**

This cross-sectional study was conducted nationwide among Chinese anesthesiologists between December 2022 and January 2023. The demographic information and KAP scores of the respondents were collected using a web-based questionnaire. The mean KAP dimension scores ≥ 60% were considered good.

**Results:**

This study enrolled 1032 anesthesiologists (51.2% male). The mean total scores of knowledge, positive attitude, and positive practice were 9.3 ± 1.2 (max 12), 34.8 ± 3.3 (max 40), and 30.6 ± 6.7 (max 40), respectively. The knowledge items with correctness scores < 60% were “the anesthetic drugs that tend to cause POCD” (23.3%) and “Treatment of POCD” (40.3%). Multivariable analysis showed that ≥ 40 years old, master’s degree or above, intermediate professional title (i.e., attending physician), senior professional title (i.e., chief physician), and working in tertiary hospitals were independently associated with adequate knowledge. Multivariable analysis showed that the attitude scores, middle professional title, and ≥ 16 years of experience were independently associated with good practice.

**Conclusions:**

These results suggest that Chinese anesthesiologists have good knowledge, favorable attitudes, and good practice toward POCD. Still, some points remain to be improved (e.g., the drugs causing POCD and managing POCD) and should be emphasized in training and continuing education.

**Trial registration:**

ChiCTR2200066749.

**Supplementary Information:**

The online version contains supplementary material available at 10.1186/s12909-024-05358-6.

## Background

Postoperative cognitive dysfunction (POCD) is characterized clinically by subtle symptom onset (typically noticed weeks to months after surgery), mild cognitive decline with improvement within weeks to months of surgery (rarely persists for years), impairment involving memory, learning, concentration, attention, and/or psychomotor performance, and alert mental status with the maintenance of orientation to person, place, and time [1]. POCD might be reversible in days to months, but if it persists beyond > 12 months postoperatively, the standard DSM 5 nomenclature is suggested (mild or major neurocognitive disorder) without the use of the “postoperative” classifier [2]. The reported incidence is 5-55% in older patients, but the wide range is due to the surgery type and the definitions used [1]. POCD may occur in patients of any age but is most common in older patients. General anesthesia exposure is associated with an increased risk of intraoperative hypotension in adults aged > 50 years [3, 4]. General and regional anesthesia might be associated with similar cognitive outcomes ≥ 7 days postoperatively in adults [5]. Bispectral index-guided anesthesia might reduce the risk of POCD [6]. Bispectral index-guided anesthesia may reduce the risk of POCD, and the type of anesthetic used may also influence the occurrence of POCD depending on the patient’s characteristics and the type of surgery [7]. Besides selecting the anesthetic agents [8, 9], effective non-pharmacological measures are available to decrease the risk of POCD and shorten, including getting the patient out of bed as soon as possible after surgery and maintaining continuous contact with other people [10]. Preventing POCD while avoiding polypharmacy and providing adequate pain control are important goals for anesthesiologists [11]. Therefore, anesthesiologists must be aware of POCD.

Knowledge, attitudes, and practice (KAP) studies are surveys designed to provide quantitative and qualitative data to identify gaps that could be barriers to a specific activity in a specific population [12, 13]. KAP studies are particularly useful for planning behavioral change interventions [12, 13]. A German study showed that the awareness of physicians toward POCD, especially persisting POCD, was low [14]. Still, the exact KAP toward POCD among Chinese anesthesiologists is unknown.

Therefore, this study aimed to investigate the KAP toward POCD among Chinese anesthesiologists and explore the factors influencing KAP.

## Methods

This cross-sectional study was conducted nationwide from December 2022 to January 2023. The inclusion criteria were (1) board-certified anesthesiologists and (2) anesthetic practice for at least 6 months. This study was ethically approved by the Ethics Committee of the Second Affiliated Hospital of Jiaxing University (approval No: 2022ZFYJ245-01). Written informed consent was obtained from each participant before he/she completed the questionnaire. This study was registered with the Chinese Clinical Trials Registry (registration No. ChiCTR2200066749). Registration link https://www.chictr.org.cn/showproj.html?proj=186869.

### Data collection

The questionnaire was designed after referring to the relevant literature [15, 16] and was revised based on the comments made by three senior experts. Two rounds of correspondence were conducted for content validity. In the first round, two anesthesiologists and one epidemiologist were selected, and according to their opinions, we added questions about POCD attitude and practice. In the second round, one anesthesiologist and one epidemiologist were selected, and the questions about POCD pathogenesis, treatment, and prevention were changed from single-choice to multiple-choice according to their opinions. Forty-three anesthesiologists were randomly selected to perform an internal consistency reliability test with a Cronbach’s α of 0.86. The questionnaires were edited and handled through the Sojump online platform (https://www.wjx.cn/app/survey.aspx). Publicity was sent to the anesthesiologists through newsletters, with a QR code to the questionnaire. The anesthesiologists interested in completing and returning the questionnaire simply had to scan the QR code. Participants were assured of anonymity during the survey process. At the beginning of the questionnaire, the respondents were asked for informed consent. If the participants had to tick “yes” to the statement “I consent to participate in this survey and to my data being used for research purposes” to access the questionnaire. Not ticking the “yes” box indicated that the participant did not consent to participate in the study and could not complete the questionnaire. Only one questionnaire could be submitted from an IP address. Incomplete questionnaires, those with obvious filling patterns (e.g., all first choices), questionnaires with logic errors, and those that took < 3 min to complete were excluded.

The final self-administered anonymous questionnaire was in Chinese and contained four dimensions: demographic information (age, gender, education, professional title, years of anesthetic practice after obtaining board certification, and hospital grade), knowledge dimension, attitude dimension, and practice dimension. Physician title is divided into four levels: primary title (physician and physician resident), intermediate title (attending physician), deputy senior title (deputy chief physician), and senior title (chief physician).

The knowledge dimension consisted of 12 questions, scored 1 point for correct answers and 0 points for incorrect or unclear answers, ranging from 0 to 12 points. The attitude dimension consisted of eight questions using a 5-point Likert scale, with positive attitude questions assigned 5 to 1 point from “Strongly Agree” to “Strongly Disagree” and negative attitude questions (items A5 and A7) were assigned points in reverse; the total score ranged from 8 to 40 points. The practice dimension contained eight questions, also using a 5-point Likert scale, ranging from “Always” (5 points) to “Never” (1 point), and ranging from 8 to 40 points. The mean KAP dimension scores ≥ 60% were considered good [17] (i.e., > 7.2 for knowledge, > 24 for attitude, and > 24 for practice). The mean values of the KAP scores were used as the cut-off values, and anesthesiologists with scores above the mean value were considered to have adequate knowledge, positive attitude, and good practice.

### Statistical analysis

The normal distribution of the continuous data was confirmed using the Kolmogorov-Smirnov test. The continuous variables were expressed as mean ± standard deviations (SD) and analyzed using Student’s t-test and ANOVA. Categorical data were expressed as n (%) and analyzed using the chi-square test. Univariable and multivariable analyses were conducted using logistic regression to analyze the factors influencing KAP. The enter method was used to screen the variables, and the variables with *P* < 0.05 in the univariable analyses were included in the multivariable analysis. All statistical analyses were performed using SPSS 26.0 (IBM, Armonk, NY, USA). Two-sided P-values < 0.05 were considered statistically significant.

## Results

### Characteristics of the participants

A total of 1092 questionnaires were received in this study. After excluding 18 participants who refused to participate in the study, 27 questionnaires had logical errors in the answers, and 15 questionnaires completed the survey within 3 min. Finally, 1032 questionnaires (1032 participants) were valid and included in the analysis. The highest frequencies of participants were observed in the following categories: 31–40 years old (40.2%), male (51.2%), with a master’s degree or above (35.2%), with a senior title (37.5%), with ≥ 16 years of experience (40.1%) and working in tertiary hospitals (72.6%) (Table 1). Among them, 537 participants were from Zhejiang, 198 were from Jiangsu, and 57 were from Shanghai (Supplementary Table S1).

**Table 1:** Characteristics of the participants

	N	%	Knowledge score		Attitude score		Practice score	
			Mean ± SD	P	Mean ± SD	P	Mean ± SD	P
**Total**			9.3 ± 1.8		34.8 ± 3.3		30.6 ± 6.7	
**Age (years)**				< 0.001		0.160		< 0.001
< 30	229	22.2	8.8 ± 2.1		34.4 ± 3.3		29.0 ± 7.9	
31–40	415	40.2	9.5 ± 1.6		34.9 ± 3.3		30.3 ± 6.4	
≥ 40	388	37.6	9.4 ± 1.8		34.9 ± 3.2		31.8 ± 6.1	
**Gender**				0.219		0.243		0.268
Male	528	51.2	9.4 ± 1.9		34.6 ± 3.3		30.8 ± 6.6	
Female	504	48.8	9.2 ± 1.7		34.9 ± 3.2		30.3 ± 6.9	
**Education**				< 0.001		0.651		0.247
Bachelor’s degree and below	669	64.8	9.2 ± 1.8		34.8 ± 3.2		30.8 ± 6.7	
Master’s degree and Above	363	35.2	9.6 ± 1.7		34.7 ± 3.4		30.3 ± 6.8	
**Professional title**				< 0.001		0.018		< 0.001
Non-title	86	8.3	8.2 ± 2.6		33.8 ± 3.5		29.7 ± 8.3	
Primary title	185	17.9	9.0 ± 1.7		34.7 ± 3.2		29.1 ± 7.4	
Middle title	374	36.2	9.3 ± 1.7		34.7 ± 3.2		30.1 ± 6.6	
Senior title	387	37.5	9.7 ± 1.7		35.0 ± 3.2		31.9 ± 5.9	
**Years of anesthetic practice after obtaining board certification**				< 0.001		0.185		< 0.001
≤ 5	218	21.1	8.8 ± 2.0		34.4 ± 3.4		28.8 ± 7.9	
5–10	200	19.4	9.5 ± 1.6		35.0 ± 3.1		29.7 ± 6.8	
11–15	200	19.4	9.4 ± 1.7		34.7 ± 3.4		30.6 ± 5.9	
≥ 16	414	40.1	9.5 ± 1.8		34.9 ± 3.2		31.9 ± 6.1	
**Hospital grade**				< 0.001		0.611		0.552
Tertiary	749	72.6	9.4 ± 1.8		34.8 ± 3.3		30.5 ± 6.8	
Non-tertiary	283	27.4	9.0 ± 1.9		34.7 ± 3.1		30.8 ± 6.4	

Physician title is divided into four levels: primary title (physician and physician resident), intermediate title (attending physician), deputy senior title (deputy chief physician), and senior title (chief physician)

### Knowledge, attitudes, and practice

The mean knowledge score was 9.3 ± 1.2 (max 12). Knowledge scores were associated with age (*P* < 0.001), education (*P* < 0.001), professional title (*P* < 0.001), experience (*P* < 0.001), and hospital level (*P* < 0.001) (Table 1). The knowledge items with correctness scores < 60% were “the anesthetic drugs that tend to cause POCD” (23.26%) and “Treatment of POCD” (40.31%). All other items were correctly answered by ≥ 60% of the participants (Table 2).

**Table 2:** Correctness of knowledge

Knowledge	Correct rate, n (%)
Multiple choice:	
Possible pathogenesis of POCD	711 (68.9)
POCD is postoperative delirium (False)	823 (79.8)
The anesthetic drugs that tend to cause POCD	240 (23.3)
Risk factors for POCD	959 (92.9)
Hazards of POCD	949 (92.0)
The central nervous system (CNS) is the target organ of almost all anesthetic and analgesic drugs, so it makes intuitive sense to evaluate the CNS before anesthetic drugs significantly alter its function	949 (92.0)
The cognitive functioning assessment scale that can be used for diagnosis in the perioperative period	967 (93.7)
Hyperventilation, hypotension, and hypoxia can cause POCD during surgery	997 (96.6)
Type of surgery does not cause POCD	912 (88.4)
The biomarkers that cause POCD	721 (69.9)
Treatment of POCD	416 (40.3)
The effective measures to prevent the development of POCD	963 (93.3)

The mean attitude score was 34.8 ± 3.3 (max 40). The attitude scores were associated with professional titles (*P* = 0.018) (Table 1). Supplementary Table S2 shows the distribution of the attitudes.

The mean practice score was 30.6 ± 6.7 (max 40). The practice scores were associated with age (*P* < 0.001), professional titles (*P* < 0.001), and experience (*P* < 0.001) (Table 1). The distribution of the practice evaluation is presented in Supplementary Table S3.

### Factors associated with knowledge, attitudes, and practice

The multivariable analysis showed that ≥ 40 years old (OR = 0.440, 95%CI: 0.201–0.963, *P* = 0.040), master’s degree or above (vs. bachelor’s degree or below, OR = 1.405, 95%CI: 1.042–1.895, *P* = 0.026), middle professional title (OR = 2.185, 95%CI: 1.009–4.733, *P* = 0.047), senior professional title (OR = 4.704, 95%CI: 1.988–11.127, *P* < 0.001), and working in tertiary hospitals (OR = 1.567, 95%CI: 1.167–2.102, *P* = 0.003) were independently associated with adequate knowledge of anesthesiologists toward POCD (Table 3). Moreover, according to the results shown in Table 4, only the knowledge score was associated with a favorable attitude in the univariable analysis (OR = 1.319, 95%CI: 1.220–1.423, *P* < 0.001). Therefore, no multivariable analysis was further conducted. Finally, the multivariable analysis revealed that good practice was independently associated with attitude scores (OR = 1.144, 95% CI: 1.098–1.192, *P* < 0.001), middle professional title (OR = 0.397, 95% CI: 0.185–0.854, *P* = 0.018), and more than 16 years of experience (OR = 2.714, 95% CI: 1.172–6.285, *P* = 0.020) (Table 5).

**Table 3:** Multivariable analysis of knowledge

Knowledge	Univariable logistic regression		Multivariable logistic regression	
	OR (95%CI)	P	OR (95%CI)	P
**Age (years)**				
< 30	REF		REF	
31–40	1.844 (1.331–2.556)	< 0.001	0.770 (0.420–1.410)	0.397
≥ 40	1.499 (1.079–2.081)	0.016	0.440 (0.201–0.963)	0.040
**Gender**				
Male	REF			
Female	0.913 (0.714–1.167)	0.466		
**Education**				
Bachelor’s degree/below	REF		REF	
Master’s degree/above	1.595(1.228–2.072)	<0.001	1.405(1.042–1.895)	0.026
**Professional title**				
Non-title	REF		REF	
Primary title	1.245 (0.7402.094)	0.410	1.252 (0.719–2.180)	0.427
Middle title	1.937 (1.201–3.125)	0.007	2.185 (1.009–4.733)	0.047
Senior title	2.760 (1.708–4.458)	< 0.001	4.704 (1.988–11.127)	< 0.001
**Years of anesthetic practice after obtaining board certification**				
≤ 5	REF		REF	
5–10	2.020 (1.368–2.985)	< 0.001	1.542 (0.828–2.872)	0.173
11–15	1.979 (1.340–2.922)	0.001	1.248 (0.604–2.578)	0.550
≥ 16	1.820 (1.306–2.535)	< 0.001	1.252 (0.526–2.980)	0.612
**Hospital grade**				
Tertiary hospitals	1.689 (1.282–2.225)	< 0.001	1.567 (1.167–2.102)	0.003
Non-tertiary hospitals	REF		REF	

**Table 4:** Univariable analysis of attitude

Attitude	Univariable logistic regression	
	OR (95%CI)	P
**Knowledge score**	1.319 (1.22–1.423)	< 0.001
**Age (years)**		
< 30	REF	
31–40	1.223 (0.88.-1.694)	0.225
≥ 40	1.124 (0.809–1.562)	0.485
**Gender**		
Male	REF	
Female	1.143 (0.893–1.464)	0.289
**Education**		
Bachelor’s degree and below	REF	
Master’s degree and above	1.030(0.795–1.335)	0.821
**Professional title**		
Non-title	REF	
Primary title	1.312 (0.786–2.192)	0.299
Middle title	1.352 (0.845–2.163)	0.208
Senior title	1.497 (0.936–2.393)	0.092
**Years of anesthetic practice after obtaining board certification**		
≤ 5	REF	
5–10	1.391 (0.940–2.058)	0.099
11–15	1.061 (0.721–1.561)	0.765
≥ 16	1.083 (0.778–1.506)	0.637
**Hospital grade**		
Tertiary hospitals	1.086 (0.824–1.432)	0.557
Non-tertiary hospitals	REF	

**Table 5:** Multivariable analysis of practice

Practice	Univariable		Multivariable	
	OR (95%CI)	P	OR (95%CI)	P
**Knowledge score**	1.052 (0.983–1.126)	0.140		
**Attitude score**	1.141 (1.096–1.187)	< 0.001	1.144 (1.098–1.192)	< 0.001
**Age (years)**				
< 30	REF		REF	
31–40	1.394 (1.007–1.930)	0.045	1.501 (0.830–2.717)	0.179
≥ 40	2.207 (1.582–3.078)	< 0.001	1.369 (0.650–2.886)	0.408
**Gender**				
Male	REF			
Female	0.828 (0.648–1.057)	0.130		
**Education**				
Bachelor’s degree and below	REF			
Master’s degree and above	0.867(0.671–1.119)	0.272		
**Professional title**				
Non-title	REF		REF	
Primary title	0.816 (0.489–1.363)	0.437	0.593 (0.340–1.035)	0.066
Middle title	1.015 (0.635–1.621)	0.952	0.397 (0.185–0.854)	0.018
Senior title	1.710 (1.069–2.737)	0.025	0.446 (0.193–1.030)	0.059
**Years of anesthetic practice after obtaining board certification**				
≤ 5	REF		REF	
5–10	1.168 (0.794–1.719)	0.430	1.299 (0.699–2.415)	0.408
11–15	1.371 (0.932–2.017)	0.109	1.605 (0.786–3.278)	0.194
≥ 16	2.269 (1.624–3.170)	< 0.001	2.714 (1.172–6.285)	0.020
**Hospital grade**				
Tertiary hospitals	0.943 (0.717–1.241)	0.675		
Non-tertiary hospitals	REF			

## Discussion

The study findings showed that Chinese anesthesiologists possess good knowledge, hold favorable attitudes, and have active practices toward POCD. However, there are areas where improvement is needed. The results of this study could be used to design training programs that will improve the KAP of anesthesiologists concerning the prevention and management of POCD.

POCD is an important medical issue in patients undergoing surgery [1, 18]. The present study showed good KAP toward POCD in Chinese anesthesiologists, while a German study showed that the awareness of POCD was higher in nurses than in physicians [14]. Similar results were observed in Sweden [19]. The present study generally showed that the knowledge of POCD improved with experience and professional title, which are often interrelated. Importantly, POCD can be prevented by non-pharmacologic approaches [10]. Still, the present study showed that Chinese anesthesiologists had poor knowledge of the drugs that cause POCD and how to manage it once it occurs. Hence, training programs will have to emphasize these two aspects in the future. Indeed, intraoperative hypotension is a clear cause of POCD [3, 4], while several anesthetics and clinical parameters are considered possible risk factors of POCD, including fentanyl, ketamine, lidocaine, magnesium sulfate infusion, piracetam, steroids, benzodiazepines, general vs. regional anesthesia, bispectral index, and monitoring based on copeptin levels, inflammatory markers, and glycemia, among others [5, 20–25]. Of note, a study in Germany showed that the implementation of mandatory training on the cognitive impacts of surgery was low [26]. In addition, existing strategies focus on screening and therapy after surgery or intensive care [19, 27], but a holistic approach appears to be missing [28]. In addition, guidelines do not address who is responsible for risk detection and communication [19, 27]. Therefore, authoritative organizations and decision-makers must also play a role in improving healthcare providers’ knowledge of POCD.

Although the survey showed that anesthesiologists have favorable attitudes and good practice toward POCD, only correct knowledge can lead to correct actions [29]. In fact, there is a lack of guidance on POCD in clinical practice, which makes anesthesiologists take different approaches toward POCD. Previous studies showed that the KAP of anesthesiologists toward POCD is low [14, 19], probably due to rare systematic POCD education and training. Therefore, some authors suggest that knowledge about perioperative brain dysfunction should be included in the basic education curriculum of anesthesiology training. Unfortunately, there are no relevant guidelines or consensuses in China. Such consensuses should be reached by experts.

In terms of training methods, anesthesiologists could use online learning software for POCD-specific knowledge through network platforms to facilitate fragmented learning, and continuous education activities could be designed and implemented. In terms of training content, the impact of anesthetic drugs on POCD and POCD treatment should be advocated as the focus of education and training, and the core principles of POCD prevention and treatment should be emphasized to improve the fundamental anesthesiologist’s management ability of POCD. The present study highlighted that the anesthesiologists had poorer knowledge regarding the drugs that can cause POCD and how to manage POCD. Such knowledge should be improved. In terms of quality control, it is suggested to attach importance to POCD management, establish relevant assessment mechanisms, and improve the standardized management level of anesthesiologists’ KAP toward POCD.

According to the KAP theory [12, 13], knowledge is the theoretical basis for practice, while attitude is the force driving practice. Still, in the present study, knowledge did not directly affect practice. Several reasons could account for that. It could be related to the way the questions are formulated. A KAP survey is a questionnaire that skims the general KAP toward a subject without going into details. It could also be because some actions are performed out of habits or according to teaching but without knowing the exact theory of why it is performed. The present study was not designed to determine why knowledge was not directly related to practice. For example, knowledge items pertaining to the drugs causing POCD and how to manage POCD showed poor knowledge about those two items, but there are no practice questions about the drugs, and the only practice question about POCD management is about referral to attending physicians and neurologists. Additional studies will be necessary to examine that issue.

A strength of this study was its nationwide nature. Still, it also had limitations. Although this study was advertised in newsletters, it was clear that older, more experienced anesthesiologists working at tertiary hospitals were enrolled, probably biasing the results. In addition, because the anesthesiologists interested in participating in the study simply had to scan the QR code, the response rate could not be calculated. Even though tertiary hospitals more frequently undertake specialized procedures than non-tertiary hospitals (primary and secondary hospitals), the emergent or routine procedures that require general anesthesia and are performed in non-tertiary hospitals also involve a risk of POCD [20]. Furthermore, KAP surveys essentially record an “opinion” based on the survey statements. Therefore, the KAP survey reveals what was said, but there is a possibility of considerable gaps between what was said and what was done.

## Conclusions

Chinese anesthesiologists have good knowledge, favorable attitudes, and active practice toward POCD. Nevertheless, some areas of KAP (e.g., the drugs causing POCD and managing POCD) were identified as needing improvements. Those areas will have to be included and highlighted in future training programs during residency or as continuing education activities.

### Electronic supplementary material

Below is the link to the electronic supplementary material.

Supplementary Material 1

## Data Availability

All data generated or analyzed during this study are included in this published article.

## Declarations

### Clinical trial number and registry URL

This study was registered with the Chinese Clinical Trials Registry (registration No. ChiCTR2200066749). Registration link https://www.chictr.org.cn/showproj.html?proj=186869.

## Abbreviations

KAP: knowledge, attitudes, and practice
POCD: postoperative cognitive dysfunction
