# Supplementary material for: Knowledge, attitudes, and practice toward postoperative cognitive dysfunction among anesthesiologists in China: a cross-sectional study
Source: BMC Med Educ. 2024 Apr 1;24:359. doi: 10.1186/s12909-024-05358-6 (PMC10986038; doi:10.1186/s12909-024-05358-6)
Supplement: Supplementary file 1 — Supplementary Material 1 [file 12909_2024_5358_MOESM1_ESM.docx]

| Province / city | **N** | % |
| --- | --- | --- |
| Anhui | **17** | 0.016 |
| Beijing | **10** | 0.010 |
| Fujian | **8** | 0.008 |
| Gansu | **1** | 0.001 |
| Guangdong | **14** | 0.014 |
| Guangxi | **2** | 0.002 |
| Guizhou | **11** | 0.011 |
| Hainan | **1** | 0.001 |
| Hebei | **9** | 0.009 |
| Henan | **11** | 0.011 |
| Heilongjiang | **10** | 0.010 |
| Hubei | **18** | 0.017 |
| Hunan | **9** | 0.009 |
| Jilin | **1** | 0.001 |
| Jiangsu | **198** | 0.192 |
| Jiangxi | **7** | 0.007 |
| Liaoning | **17** | 0.016 |
| Inner Mongolia | **8** | 0.008 |
| Ningxia | **4** | 0.004 |
| Shandong | **23** | 0.022 |
| Shanxi | **9** | 0.009 |
| Shaanxi | **19** | 0.018 |
| Shanghai | **57** | 0.055 |
| Sichuan | **11** | 0.011 |
| Taiwan | **1** | 0.001 |
| Tianjin | **1** | 0.001 |
| Tibet | **1** | 0.001 |
| Xinjiang | **6** | 0.006 |
| Yunnan | **4** | 0.004 |
| Zhejiang | **537** | 0.520 |
| Chongqing | **5** | 0.005 |
| Abroad | **2** | 0.002 |

**Supplementary Table S1.** Region of the participants

**Supplementary Table S2.** Distribution of attitude (%)

|  | Strongly agree | Agree | Neutral | Disagree | Strongly disagree |
| --- | --- | --- | --- | --- | --- |
| Advocacy and education regarding POCD is necessary for patients and their families. | 85.08 | 13.37 | 1.16 | 0.29 | 0.10 |
| The possible risks of POCD should be explained in detail to the patient before surgery | 78.88 | 17.34 | 2.52 | 1.07 | 0.19 |
| The anesthesiologist should also follow up with the patient after surgery to understand the changes in cognitive function | 82.56 | 15.41 | 1.74 | 0.10 | 0.19 |
| Preoperative related precautions can help avoid POCD | 63.47 | 24.52 | 9.21 | 2.62 | 0.19 |
| Even if POCD is treated accordingly, it cannot be cured | 18.60 | 17.34 | 18.90 | 34.88 | 10.27 |
| Cognitive assessment of the patient before anesthesia is a must-do | 68.22 | 26.94 | 4.36 | 0.48 | 0.00 |
| The diagnosis of POCD should be made by the patient’s attending physician, not the anesthesiologist | 13.18 | 8.53 | 16.09 | 43.31 | 18.90 |
| Anesthesiologists need to be well informed about POCD | 81.01 | 17.83 | 0.97 | 0.19 | 0.00 |

**Supplementary Table S3.** Distribution of practice (%)

|  | Always | Often | Sometimes | Occasionally | Never |
| --- | --- | --- | --- | --- | --- |
| I will assess the patient’s cognitive function prior to anesthesia | 30.72 | 32.17 | 26.84 | 7.95 | 2.33 |
| I will preoperatively adjust the status of patients with risk factors for POCD to prevent POCD | 32.07 | 34.21 | 23.16 | 8.14 | 2.42 |
| I will take various means during the surgery to prevent POCD and to protect the brain function of patients | 43.80 | 38.47 | 14.05 | 2.81 | 0.87 |
| I will follow up with patients after surgery to assess their cognitive status | 30.14 | 32.46 | 23.26 | 12.21 | 1.94 |
| I will inform the patient’s family to keep an eye on the patient’s brain function and cognitive function after surgery | 31.69 | 29.75 | 22.38 | 11.63 | 4.55 |
| I will inform the patient’s attending physician to keep an eye on the patient’s brain function and cognitive function after surgery | 30.33 | 28.10 | 25.68 | 12.02 | 3.88 |
| I will take the initiative to learn about POCD or work on POCD research | 30.23 | 31.59 | 27.91 | 9.21 | 1.07 |
| When a patient is found to have postoperative POCD, I will discuss with the attending physician and neurologist to propose an effective treatment plan | 30.43 | 29.46 | 25.19 | 10.37 | 4.55 |
